# Severe Pneumonia Associated with Adenovirus Type 55 Infection, France, 2014

**DOI:** 10.3201/eid2211.160728

**Published:** 2016-11

**Authors:** Jérémy Lafolie, Audrey Mirand, Maud Salmona, Alexandre Lautrette, Christine Archimbaud, Amélie Brebion, Christel Regagnon, Martine Chambon, Séverine Mercier-Delarue, Jérôme Le Goff, Cécile Henquell

**Affiliations:** Centre Hospitalier Universitaire Gabriel Montpied, Clermont-Ferrand, France (J. Lafolie, A. Mirand, A. Lautrette, C. Archimbaud, A. Brebion, C. Regagnon, M. Chambon, C. Henquell);; Université d’Auvergne, Clermont-Ferrand (J. Lafolie, A. Mirand, A. Lautrette, C. Archimbaud, M. Chambon, C. Henquell);; Hôpital Saint-Louis, Paris, France (M. Salmona, S. Mercier-Delarue, J. Le Goff);; Université Paris Diderot, Paris (M. Salmona, J. Le Goff)

**Keywords:** adenovirus, pneumonia, immunocompetence, viruses, France, respiratory infections

**To the Editor:** Human adenoviruses (HAdVs) comprise 70 recognized genotypes (as of February 15, 2016; http://hadvwg.gmu.edu/) and are frequently associated with mild and acute upper respiratory tract infections, depending on virus type and host immune status (*1*). HAdV type 55 (HAdV-55) has recently reemerged as a highly virulent pathogen, causing severe and sometimes fatal pneumonia among immunocompetent adults, particularly in Asia (*2**–**4*). Formerly known as HAdV-11a, HAdV-55 is a genotype resulting from recombination between HAdV-11 and HAdV-14 (*5*). We report 2 cases of severe pneumonia associated with HAdV-55 infection in France.

In November 2014, two immunocompetent women, 71 (patient A) and 36 (patient B) years of age, sought care 4 days apart at the emergency unit of the University Hospital of Clermont-Ferrand, France, for an influenza-like syndrome characterized by fever, cough, and dyspnea. Laboratory investigations at admission revealed thrombocytopenia (98 and 88 × 10^9^ thrombocytes/L for patients A and B, respectively; reference range 150–450 × 10^9^ thrombocytes/L) and elevated C-reactive protein concentrations (71.6 and 45.8 mg/L, respectively; reference range <3 mg/L). Chest radiographs and thoracic tomodensitometry images showed acute left lobar pneumonia in each patient. Therapy with intravenous antimicrobial drugs (cefepime and levofloxacin) and oxygen was initiated. Patient A was transferred to the intensive care unit 4 days after admission because of unimproved respiratory function; patient B was transferred 5 days after admission because of acute respiratory distress syndrome. 

Results for all bacteriologic analyses were negative (blood cultures, bronchoalveolar lavage fluid cultures, PCR for *Mycobacterium tuberculosis* [Xpert MTB/RIF; Cepheid, Sunnyvale, CA, USA] of bronchoalveolar lavage fluid, and urinary antigen testing [BinaxNOW *Legionella* and *Streptococcus pneumoniae*; Alere, Scarborough, ME, USA]). No specific antibodies were detected against *Chlamydia pneumoniae* (Anti-*C. pneumoniae*; Euroimmun, Lübeck, Germany) and *Mycoplasma* *pneumoniae* (Platelia *M. pneumoniae* IgM; Bio-Rad, Hercules, CA, USA). For each patient, HAdV was the only pathogen detected in nasopharyngeal secretions collected at admission and in bronchoalveolar lavage fluids collected while in the intensive care unit (molecular multiplex assay [FilmArray Respiratory Panel; bioMérieux, Durham, NC, USA]). HAdV DNA was also detected in whole blood (Adenovirus R-gene; bioMérieux); viral load was 280,524 copies/mL for patient A 9 days after hospital admission and 951,146 copies/mL for patient B 4 days after admission. During hospitalization, transient hepatitis developed in each patient; serum aspartate aminotransferase levels were elevated up to 6–10 times reference range, and leukocyte counts indicated leukopenia (2.17 and 1.28 × 10^9^/L for patients A and B, respectively; reference range 4–10 × 10^9^/L). Patient B had acute pancreatitis and hyperlipasemia (lipase 1,697 UI/L; reference range 73–393 UI/L). Healthy respiratory function was restored for both patients, who were discharged 26 (patient A) and 19 (patient B) days after admission.

A partial region of the hexon gene was amplified and sequenced from DNA extracts of respiratory and blood samples, as previously described (*6*). Phylogenetic analysis with strains representing all HAdV genotypes identified the viruses as HAdV-55 (data not shown). We performed complete-genome sequencing, which is now recommended for confirmation of HAdV type, by using next-generation sequencing from blood samples (Technical Appendix). Genome coverage (34,755 nt) was 99.1% (patient A) and 96.1% (patient B). Phylogenetic analysis showed that the sequences of the isolates from the 2 patients clustered together (bootstrap 99%) and were genetically more closely related to the sequences of the CQ-814 strain isolated in China in 2010 and the strain from Argentina (GenBank accession no. JX423384) (Figure, panel A). To investigate genetic relationships with more strains from distant geographic areas, we performed phylogenetic analyses with all available sequences of the hexon gene of HAdV-55 strains. However, because diversity of this gene between strains was low, we could not determine the geographic origin of the strains from France, which were genetically distant from the strain isolated in Spain in 1969 (Figure, panel B).

**Figure:**
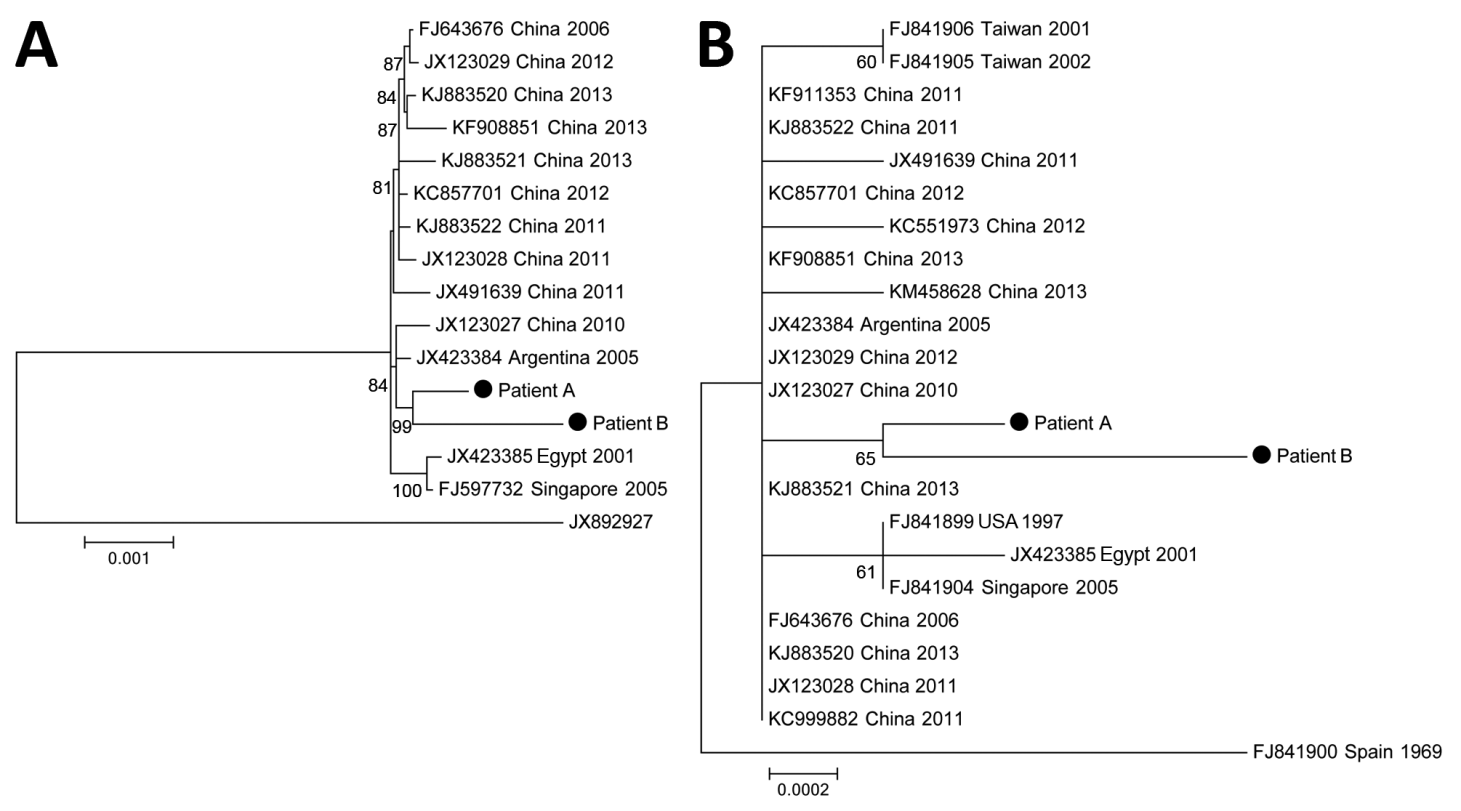
Phylogeny of 13 complete-genome sequences of human adenovirus type 55 (A) and of 21 sequences of hexon genes (B). The complete genome tree (A) is rooted to a human adenovirus type 14 isolate (GenBank accession no. JX892927). The strains from patients A and B (immunocompetent women with human adenovirus infection) reported in this study are indicated. The phylogenetic tree was calculated by using the maximum-likelihood method in MEGA6 (http://www.megasoftware.net). The best algorithm was chosen by the criterion score of the Bayesian information criteria. The statistical robustness of branches was estimated by 1,000 bootstraps. Only bootstrap values >70% are indicated. The tree is drawn to scale; branch lengths are measured in number of substitutions per site (scale bar). All positions containing gaps and missing data were eliminated. The sequence of the hexon gene from patient B was partially complete (2,821/2,841, 99.3%).

Over the past 10 years, reports of HAdV-55 have been increasing in Asia during outbreaks of respiratory diseases that in some cases led to severe pneumonia and deaths in immunocompetent adults and children (*2**–**4**,**7**,**8*)*.* Of the 969 cases of community-acquired pneumonia in adults, 48 (5%) were associated with HAdVs; HAdV-55 was identified in 21 (43.8%) of these patients (*7*). For the 2 patients we report, clinical features were similar to those described elsewhere (*4**,**8*). Neither patient had traveled recently, and the 2 patients had not had contact with each other. Analysis of complete genomic sequences showed that the viruses infecting the patients were distinguishable from strains previously isolated in other countries. HAdV-55 could thus have been circulating in France for several years. Since its first detection in Spain in 1969 (*9*), HAdV-55 has been reported only 1 time in Europe, in Germany in 2004 (*10*). 

Because most HAdV infections are asymptomatic and respiratory virus screening in routine practice does not systematically include HAdV detection, the true prevalence and clinical effect of HAdV-55 infection has probably been underestimated. The involvement of virus of this genotype in severe pneumonia emphasizes the need to reinforce HAdV surveillance by including HAdV genome detection and genotyping (if positive) in the documentation of severe respiratory infections.

Technical Appendix Full-genome sequencing and analysis of human adenovirus type 55.
