# Supplementary material for: Severe Pneumonia Associated with Adenovirus Type 55 Infection, France, 2014
Source: Emerg Infect Dis. 2016 Nov;22(11):2012–4. doi: 10.3201/eid2211.160728 (PMC5088017; doi:10.3201/eid2211.160728)
Supplement: Technical Appendix — Full-genome sequencing and analysis of human adenovirus type 55. [file 16-0728-Techapp-s1.pdf]

Article DOI: http://dx.doi.org/10.3201/eid2211.160728

Severe Pneumonia Associated with Adenovirus Type 55, France, 2014

Technical Appendix

HAdV Full-Genome Sequencing and Analysis

Because HAdV-55 isolation on A549 cells was unsuccessful for both patients, the full genome sequence was assessed directly from clinical samples by a metagenomic approach with the MiSeq Reagent kit v2 on a Miseq 2000 Illumina system (Illumina, San Diego, CA). Three different strategies were used and combined to obtain maximum genome coverage. Except for nucleic acid purification steps, libraries were generated as previously described (Naccache S. et al., Genome Res 2014) with Nextera XT DNA Sample Preparation Kit (Illumina). Libraries were sequenced on an Illumina MiSeq 2000 according to standard Illumina protocols, creating 250-nt paired-end reads. 1) Total nucleic acids from plasma samples were extracted with EasyMag® (bioMérieux). 2) To remove most of the host DNA and thus to enrich the viral sequences, plasma samples were first incubated with Turbo DNASe (Ambion). We then followed the protocol mentioned above. 3) After extraction of total nucleic acids from plasma samples as described in 1), host DNA was removed by MBD2-Fc-bound magnetic Bead and the remaining nucleic acids were purified with the Zymo DNA Clean and Concentrator kit.

For all MiSeq runs, after a quality filtering step with FastQ Quality Trimmer tool implemented on Galaxy (Blankenberg D. et al., Bioinformatics 2010), reads were aligned on HADV-55 JX491639 genome by Bowtie 2 (Langmead B. et al., Nat methods 2012). As no approach managed to obtain sufficient coverage of the reference genome, aligned reads of the three strategies were combined by Geneious R8 (Biomatters) (Kearse M. et al., Bioinformatics 2012) to process a reference-based assembly.
